# GITRL-armed Delta-24-RGD oncolytic adenovirus prolongs survival and induces anti-glioma immune memory

**DOI:** 10.1093/noajnl/vdz009

**Published:** 2019-06-05

**Authors:** Yisel Rivera-Molina, Hong Jiang, Juan Fueyo, Teresa Nguyen, Dong Ho Shin, Gilbert Youssef, Xuejun Fan, Joy Gumin, Marta M Alonso, Sheetal Phadnis, Frederick F Lang, Candelaria Gomez-Manzano

**Affiliations:** 1 Department of Neuro-Oncology, The University of Texas MD Anderson Cancer Center, Houston, Texas; 2 Department of Neurosurgery, The University of Texas MD Anderson Cancer Center, Houston, Texas; 3 Department of Pediatrics, The University of Texas MD Anderson Cancer Center, Houston, Texas; 4 MD Anderson Cancer Center UT Health Graduate School of Biomedical Sciences, Houston, Texas; 5 Department of Pediatrics, Clinica Universidad de Navarra, Pamplona, Spain; 6 Program in Solid Tumors, Center for the Applied Medical Research (CIMA), University of Navarra, Pamplona, Spain

**Keywords:** Delta-24, GITRL, glioblastoma, oncolytic adenovirus, viroimmunotherapy

## Abstract

**Background:**

Viroimmunotherapy is evolving as a strong alternative for the standard treatment of malignant gliomas. Promising results from a recent clinical trial testing the anticancer effect of Delta-24-RGD in patients with glioblastoma suggested the induction of antitumoral immunity after viral administration. To further enhance the anti-glioma immune effect, we have armed Delta-24-RGD with the costimulatory ligand GITRL (Delta-24-GREAT [Glucocorticoid Receptor Enhanced Activity of T cells]).

**Methods:**

We tested the infectivity and replication of Delta-24-GREAT, and the expression of ectopic GITRL in human and murine glioma cell lines. In vivo experiments involved the intracranial implantation of glioma cells into an immunocompetent model to study the anticancer effect, and rechallenging experiments to study long-term protection. Phenotypic and functional characterization of lymphocyte populations were performed by FACS and ELISA for Th1 cytokines expression, respectively.

**Results:**

Our results showed that Delta-24-GREAT infects and induces the expression of GITRL. Delta-24-GREAT prolonged the survival of glioma-bearing immunocompetent mice and resulted in both anti-viral and anti-glioma immune responses, including increased frequency of central memory CD8^+^ T cells. Rechallenging the surviving mice with a second implantation of glioma cells did not lead to tumor growth; however, the surviving mice developed lethal tumors when B16/F10 melanoma cells were implanted intracranially, strongly indicating that the immune response was specific for glioma antigens.

**Conclusions:**

GITRL-armed Delta-24-RGD treatment results in an antigen-restricted antitumor memory, an enhanced anti-glioma effect, and the generation of central immune memory. Our results strongly indicate that this strategy represents a vertical advance in virotherapy designed to treat patients with malignant brain tumors.

**Key points**

1. GITRL-armed Delta-24-RGD treatment results in enhanced anti-cancer effect and long-term survivors.
2. Delta-24-GREAT enhanced recruitment and activation of T cells in gliomas.
3. Delta-24-GREAT generates antigen-restricted anti-tumor central memory.

**Importance of the Study**

The translation of the virotherapy to the clinical setting has resulted in encouraging results for the treatment of patients with cancer. To increase the percentage of responders to viroimmunotherapy, we decided to arm Delta-24-RGD with a GITRL expression system with the goal of activating the T-cell population recruited to the tumors after viral infection. Our data showed the improved anti-glioma effect of the armed oncolytic adenovirus, Delta-24-GREAT, and the increased frequency and activation of T cells. Furthermore, rechallenging experiments showed a specific antitumoral immunity together with amplified central T-cell memory. This study encourages the preclinical testing of the next generation of oncolytic adenoviruses armed with regulators of the immune checkpoints. This vertical improvement in the virotherapy design is associated with an enhanced immune response against the tumor and the establishment of central immune memory, suggesting the possibility of improving the response rate to virotherapy in future clinical trials.

Viroimmunotherapy strategy may represent a vertical improvement in the treatment of patients with solid tumors. Approval of the oncolytic virus talimogene laherparepvec (T-VEC) by the US Food and Drug Administration for the treatment of melanoma has opened a new path for other oncolytic virus pipelines.^1^Our approach to treat gliomas involves an evolving platform of replication-competent adenoviruses targeted to the Rb pathway to generate tumor-selectivity.^2^ The second generation of these therapeutic agents, Delta-24-RGD,^3^ was successfully translated to the clinical settings^4^ (ClinicalTrials.gov ID: NCT00805376) and is currently being tested in Phase I clinical studies in several institutions in the United States and Europe for the treatment of adult and pediatric patients suffering from recurrent glioblastoma and diffuse intrinsic pontine gliomas, respectively (ClinicalTrials.gov ID: NCT02798406; NCT03178032). Results from a completed Phase I clinical trial to treat patients with recurrent malignant gliomas with Delta-24-RGD showed complete or partial responses in 20% of the patients, with five patients surviving more than 3 years.^4^ Examination of human surgical specimens 2 weeks after infection showed intratumoral viral replication and subsequent oncolytic effect. In addition, in a subset of patients, periodic magnetic resonance imaging (MRI) demonstrated the presence of pseudoprogression preceding the responses. Consistent with the radiological data, histopathological analyses showed increased T-cell infiltration and Th1 responses. Previous results from a phase I trial using an E1B-attenuated adenovirus, ONYX-015,^5^ and recent data using other oncolytic viruses, such as T-VEC and poliovirus,^6,7^ also suggest the development of an antitumoral immune response that will be eventually responsible for the eradication of the tumor. This paradigm shift in the interpretation of virotherapy implies that enhancement of the immune arm of the treatment will result in more potent and efficacious oncolytic viruses.

Agonistic treatments targeting costimulatory tumor necrosis factor receptor superfamily (TNFRSF) have been shown to enhance the proliferation and activation of T cells.^8,9^ One key member of the TNFRSF, glucocorticoid-induced TNFR family-related gene (GITR) is expressed in Tregs, naïve T cells, NK cells, and is upregulated on most of immune cell types upon activation.^9,10^ The ligand of GITR, GITRL, is widely expressed in the immune system, mainly in antigen-presenting cells (APCs).^9,10^ GITR offers a costimulatory signal to both CD4^+^ and CD8^+^ antigen-primed T cells enhancing their proliferation and their effector function.^11^ GITR/GITRL agonist antibodies have shown potent tumoricidal activity in preclinical studies targeting several solid tumors, including melanoma, sarcoma, colon, and urothelial cancers,^12^ which led to phase I clinical trials with no significant toxicity.^8^ Unlike antibodies, costimulatory ligands can be easily incorporated into replication-competent oncolytic adenoviruses. Infection of cancer cells with these armed viruses would induce the expression of the costimulatory molecule on their cell membranes that would directly interact with the tumor-infiltrating lymphocytes to amplify and enhance the antitumor T-cell activity.

Here, we present the generation and testing of an oncolytic adenovirus armed with a positive activator of the immune synapsis GITRL/GITR. Delta-24-RGD has been modified genetically to express the murine form of GITRL in order to facilitate the immune co-activation within the tumor microenvironment. Our data showed the improved anti-glioma effect of the armed oncolytic adenovirus, Delta-24-GREAT (Glucocorticoid Receptor Enhanced Activity of T cells), and the increased frequency and activation of T cells. Furthermore, rechallenging experiments showed a specific antitumoral immunity together with amplified central T-cell memory.

## Materials and Methods

### Cell Lines

Murine glioma GL261 cells (NCI-Frederick Cancer Research Tumor Repository), GL261-5 cells (isolated GL261 clone with slower in vivo growth kinetics),^13^ human gliomas U-87 MG and U-251 MG, and lung carcinoma A549 cells (ATCC), were cultured in Dulbecco’s modified Eagle’s medium-nutrient mixture F12 (DMEM/F12). GSC17, characterized previously,^14^ were maintained in DMEM/F12 supplemented with B27 (Life Technologies), epidermal growth factor and basic fibroblast growth factor (bFGF) (20 ng/mL, each) (Sigma-Aldrich). Mouse melanoma cell line B16-F10 (ATCC) was maintained in RPMI 1640 medium. Mouse glioma CT-2A (kindly donated by Dr. Thomas Seyfried, Boston College, MA)^15^ and human embryonic kidney 293 (Qbiogene, Inc.) were maintained in DMEM. Cultures, except for GSC17, were supplemented with 10% fetal bovine serum (HyClone Laboratories), and antiobiotics (100 μg/mL penicillin; 100 μg/mL streptomycin) (Corning).

### Adenoviruses

To generate Delta-24-GREAT, the murine GITRL (mGITRL) (InvivoGen) was subcloned into the KpnI/XhoI site in pcDNA3.1(+) (Life Technologies); then, the expression cassette for mGITRL (including the cytomegalovirus promoter and bovine growth hormone polyadenylation sequences) was subcloned into the ClaI/BamHI site (in place of the *E3* region) of pAB26-RGD,^3^ producing pAB26-RGD-mGITRL. The final adenoviral genome was generated by homologous DNA recombination of pAB26-RGD-mGITRL and SwaI-linearized pVK500C.Delta-24 in *Escherichia coli*, BJ5183 (2, 3). To rescue the Delta-24-GREAT (Delta24-RGD-mGITRL) virus, the resulting viral backbone vector was digested with *Pac*I and then transfected into 293 cells with X-tremeGENE HP DNA transfection reagent (Roche Diagnostics Corporation). Thus, the resulting virus, Delta-24-RGD-GREAT, contained the following modifications: replacement of the *E3* region of the human adenovirus type 5 (hAd5) genome with an mGITRL expression cassette; deletion of 24 base-pairs in the *E1A* gene; and insertion of an RGD-4C motif-coding sequence in fiber gene.^2,3^ The modification of the viral genome was confirmed through amplification of the modified region by polymerase chain reaction and then by sequencing the products. The replication-competent viruses were propagated in A549 cells, purified by the Adenopure kit (Puresyn, Inc.), and stored at −80°C. Delta-24-RGD construction was previously reported.^3^ Delta-24-GREAT replication was inactivated (UV-inactivated virus) by exposure to seven cycles of 125 J UV light in a GS Gene Linker UV Chamber (Bio-Rad)

Viral titer and replication were determined by measuring infectious units per mL (ifu/mL), following a previously published protocol.^16^ Briefly, 293 cells were incubated in 24-well plates with serial dilutions of the viral stock. Forty-eight hours later, cultures were fixed with 100% ice-cold methanol for 10 minutes at −20°C. Cells were stained for hexon expression, using an anti-adenovirus polyclonal antibody (1 h), followed by secondary staining with a biotinylated anti-goat IgG (1 h). The Vector Vectastain ABC kit (PK-4000) and ImmPACT DAB Peroxidase substrate kit (SK-4105-Reagent) were used for visualization of positive cells (Table 1 shows antibodies and working dilutions). Hexon stained areas were counted under a light microscope (20× objective) in 10 individual fields per well. In wells with viral dilutions showing 5–50 positive cells/field, the viral titer was calculated using the following formula: infectious units/mL (ifu/mL) = [(average positive cells/field) * (fields/well)] / [volume virus (mL) * dilution factor].

**Table 1.** Antibodies and Their Conditions Used for Each Assay

Experiment	Antibody	Clone	Dilution	Company	Catalog No.
Adenovirus infectious assay					
	Adenovirus hexon		1:500	Millipore	AB1056
	(Biotinylated anti-goat IgG (H+L)		1:500	Vector Lab	BA-5000
Western blot					
	Adenovirus fiber	4D2	1:1000	Thermo Fisher Scientific	MS-1027-P
	Anti-mouseGITRL/TNFSF18	721926	1:250	R&D Systems	MAB21772
	Anti-E1A (Ad2/Ad5)	M73	1:1000	EMD Millipore	05-599
	α-Tubulin	B-5-1-2	1:1000	Santa Cruz	sc-23948
	GAPDH	6C5	1:1000	Santa Cruz	sc-32233
	Goat anti-mouse IgG (H+L), HRP		1:1500	Thermo Fisher Scientific	32430
	Goat anti-rat IgG (H+L), HRP		1:10 000	Thermo Fisher Scientific	31470
Flow cytometry					
	Anti-mouse CD8a PE	53–6.7	1:100	eBiosciences	12-0081-82
	Anti-mouse CD4 eFluor® 450	GK1.5	1:100	eBiosciences	48-0041-82
	Anti-mouse CD3e FITC	145-2C11	1:100	eBiosciences	11-0031-85
	Anti-mouse CD45 APC efluor 780	30-F11	1:100	eBiosciences	47-0451-82
	CD16/CD32, rat anti-mouse	2.4G2	1:100	BD Biosciences	BDB553141
	Alexa Fluor® 647 rat anti-mouse GITRL	MIH44	1:100	BD Biosciences	563542
	Alexa Fluor® 647 rat anti-mouse GITR	DTA-1	1:100	BD Biosciences	565151
	Alexa Fluor® 647 rat IgG2b κ isotype	A95-1	1:100	BD Biosciences	557691
	PE anti-mouse CD62L	MEL-14	1:100	BioLegend	104407
	Brilliant Violet 605™ anti-mouse/human CD44	IM7	1:100	BioLegend	103047

### Western Blots

To detect protein expression, cell lysates were prepared using RIPA lysis buffer (20-mM HEPES, pH 7.0, 200-mM NaCl, 1-mM EDTA, 1-mM EGTA, 1% Triton X-100, 5-mM sodium pyrophosphate, 80-mM β-glycerophosphate, 50-mM NaF, 0.1% SDS) plus freshly added protease inhibitor cocktail (1×, Sigma-Aldrich), proteasome inhibitor (MG-132, 1 µM, Calbiochem), and phosphatase inhibitor cocktail 3 (2.5 mg/mL, Sigma-Aldrich). Cell lysates were sonicated on ice at 30% amplitude (turned on for 5 s and turned off for 45 s) to complete four cycles, and centrifuged for 10 minutes at 4°C, 13 000 rpm. Supernatants were collected, and the concentration of protein was measured using Bradford Protein reagent (Bio-Rad). DTT (50 mM) and NuPAGE LDS sample buffer (Thermo Fisher Scientific) were added to 30 μg of total protein. After being heated at 95°C for 5 minutes, samples were run in 4%–20% Novex Tris-Glycine gels (Invitrogen), and then transferred to a PVDF membrane (Thermo Fisher Scientific) and probed with antibodies (Table 1 shows antibodies and working dilutions). Protein bands were visualized using ECL western blot detection system (Amersham Pharmacia Biotech).

### In Vivo Studies

For tumor implantation, 5 × 10^4^ GL261-5 cells were implanted on day 0 of the experiment into the brains of 7- to 10-week-old C57BL/6 mice using a guide-screw system as previously described.^3^ Then, mice were randomly assigned to experimental groups. Adenoviruses (5 × 10^7^ ifu/dose) were injected intratumorally on days 6, 8, and 10 of the experiment. Mice surviving more than 100 days were rechallenged with a new intracranial tumor injection using GL261-5 (5 × 10^4^ cells/mouse) or B16-F10 (1× 10^3^ cells/mouse) cells. All experimental procedures involving the use of mice were done in accordance with protocols approved by the Animal Care and Use Committee of MD Anderson Cancer Center and National Institutes of Health and United States Department of Agriculture guidelines.

### Flow Cytometry Analysis

To analyze cell surface protein expression, cells (3 × 10^5^) were first incubated in 100 μL of primary antibody solution diluted in PBS plus 3% BSA and 1 mM EDTA. After incubation at 4°C in the dark for 30 minutes, the cells were washed once with 1 mL cold PBS. If a secondary antibody was to be applied, the incubation procedure was repeated. The cells were finally resuspended in 0.5 mL PBS. The disruption of the cell membrane upon viral infection was detected by using 8 μM ethidium homodimer 1 (Sigma-Aldrich) in PBS for 15 minutes at room temperature before acquiring the cells events. The stained cells were then acquired using the flow cytometer FACS Calibur (BD Biosciences), Gallios (Beckman-Coulter), or BD Celesta (BD Biosciences). FlowJo V10 (FlowJo, LLC) was used for the analysis. Table 1 shows the antibodies and working conditions used.

### Brain Histopathology

Paraffin-embedded sections of the mouse brain tumors were deparaffinized and rehydrated with xylene and ethanol following conventional procedures. Tumor sections were stained with Harris Hematoxylin (Fisher Scientific) and Eosin-Y solution (Fisher Scientific), and mounted with Cytoseal 60 (Thermo Fisher Scientific). Images were captured using a bright-field microscope (Zeiss Axioskop 40).

### Preparation of Cell Suspension From Murine Brains, Splenocytes, and Lymph Nodes

Mouse cerebral hemispheres bearing tumors, splenocytes, and axillary and cervical lymph nodes were collected. Initial suspensions were obtained using 100-μm cell strainers (Fisher Scientific), which were placed in RPMI 1640 medium (5 mL/sample), gently pipetted up and down, filtered through 40-µm cell strainers (Fisher Scientific), and then were pelleted by centrifugation (350 g for 7 min, RT). Specifically, for the brain tissue, it was digested using the Brain Tumor Dissociation Kit (P) (Miltenyi Biotech) as recommended by the manufacturer. The spleen-derived pellet was resuspended in Red Blood Cell Lysing Buffer Hybri-Max (Sigma-Aldrich) to lyse the red blood cells, according to the manufacturer’s instructions; then, it was brought up to 2 mL/sample with RPMI 1640. The cells from the brains, spleens, and lymph nodes were pelleted by centrifugation at 350 g for 7 minutes at room temperature and finally resuspended in PBS with 1 mM EDTA.

### Analysis of the Stimulation of Splenocytes in Cocultures With Target Cells

2 × 10^4^ target GL261-5 cells/well were seeded on 96 well-round bottom dishes (VWR) and infected with virus at 100 ifu per cell. Four hours later, 100 units/mL of mouse IFNγ (ProSpec) were added to the culture. Forty-eight hours after viral infection, the cells were detached with 2 mM EDTA in PBS, fixed with 1% paraformaldehyde, then cleaned with Lysine (0.1 M) wash solution. 2 × 10^4^ fixed cells were seeded to 96 well-round bottom dishes (VWR). To activate immune cells, pre-fixed target cells were incubated with splenocytes (5 × 10^5^/well) for 48 hours. Then, the concentration of IFNγ in the supernatant was assessed with standard ELISA (IFN-gamma DuoSet ELISA, R&D system).

### Statistical Analyses

In quantitative studies using cultured cells, each tested group consisted of triplicate samples, and experiments were performed at least by duplicate. The differences between groups were evaluated using a 2-tailed Student’s *t*-test. The animal survival curves were plotted according to the Kaplan–Meier method. Survival rates in the different treatment groups were compared using the log-rank test. *P* values < .05 were considered as significant.

## Results

### Armed Delta-24-RGD Oncolytic Adenovirus Induces mGITRL Expression on the Surface of Glioma Cells

**Figure 1.**
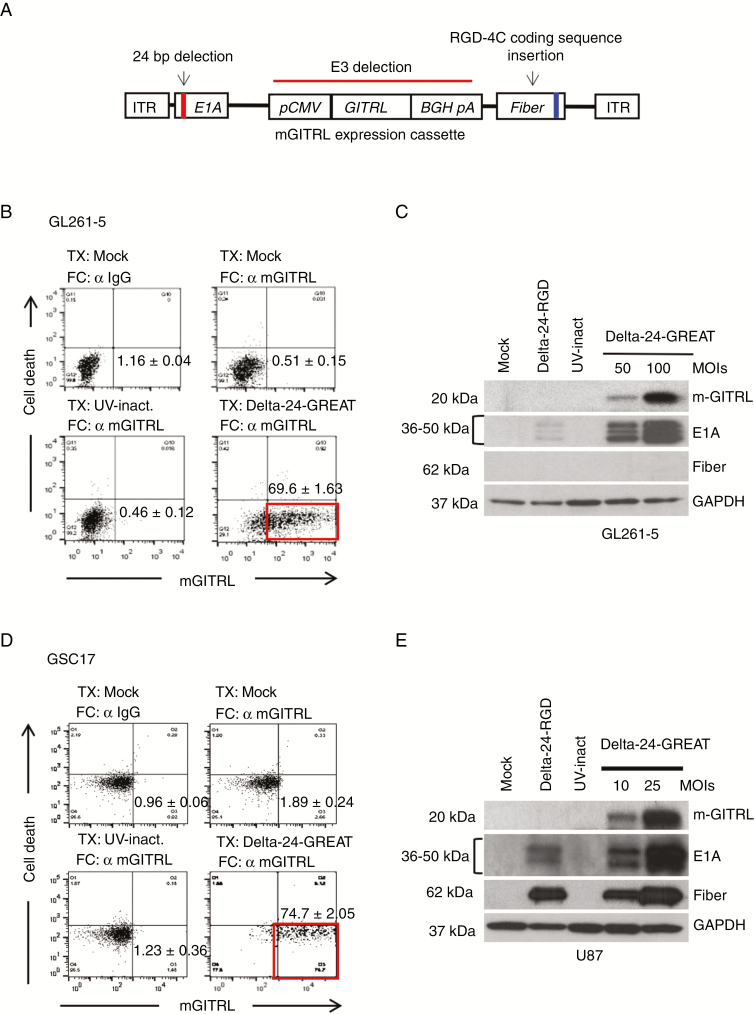
Generation of Delta-24-GREAT. (**A**) Schematic representation of Delta-24-GREAT genomic structure. *pCMV*, cytomegalovirus promoter; *BGH pA*, bovine growth factor polyadenylation signal; *ITR*, inverted terminal repeat. (**B** and **D**) Expression in vitro of the GITR ligand (GITRL) on the surface of Delta-24-GREAT-infected murine glioma cells GL261-5 (100 MOIs) (B) and human brain tumor stem cells GSC17 (25 MOIs) (**D**) assessed by FACS, 48 hours after infection. Mock-infected cells stained with IgG isotype as primary antibody, and UV-inactivated Delta-24-GREAT-infected cells were used as control. Non-viable cells were excluded from the analysis using ethidium homodimer. Data are shown as mean ± SD of three independent experiments. α, antibody. (C and **E**) Expression of viral proteins, E1A and fiber, and murine GITRL, assessed by western blotting, in Delta-24-GREAT-infected murine glioma GL261-5 cells (**C**) and human glioma U-87MG cells (E) 48 hours after infection at the indicated MOIs. Mock, Delta-24-RGD, and UV-inactivated Delta-24-GREAT were used as controls at the highest MOI. GAPDH expression was used as loading control.

We have modified the Delta-24-RGD oncolytic adenovirus to express the immune checkpoint GITRL to generate Delta-24-GREAT. The E3 viral genomic region of Delta-24-RGD was replaced by an expression cassette containing the mouse GITRL (mGITRL) cDNA (Figure 1A). The armed oncolytic adenovirus maintains the genomic modifications that secure both an enhanced infection (insertion of an RGD-4C coding region in the HI loop of the fiber) and selective replication in cancer cells (24-bp deletion of E1A)^2,3^ (Figure 1A). GL261-5 and CT-2A murine glioma cells, U-87 MG, U-251 MG human glioma cells, and GSC17 brain tumor stem cells, were efficiently infected, and the cell cultures expressed mGITRL on the surface of 65%–80% of cells 48 hours after infection (*P* < .001 when compared to uninfected cells, Student’s *t*-test) (Figure 1B and D; Supplementary Figure 1B and C). Infected human glioma cultures expressed the viral proteins E1A and fiber, as a sign of efficient infection and viral replication, respectively (Figure 1E, Supplementary Figure 1D and E). As expected, although early viral protein E1A was expressed in murine cells,^13^ viral replication and fiber expression were partially compromised (Figure 1C, Supplementary Figure 1A and E). These results confirmed the efficiency of the armed oncolytic adenovirus in inducing the ectopic GITRL expression on the membrane of glioma cells in vitro.

### GITRL Expression of Glioma Cells Enhances Delta-24-RGD-Mediated Anticancer Effect

**Figure 2.**
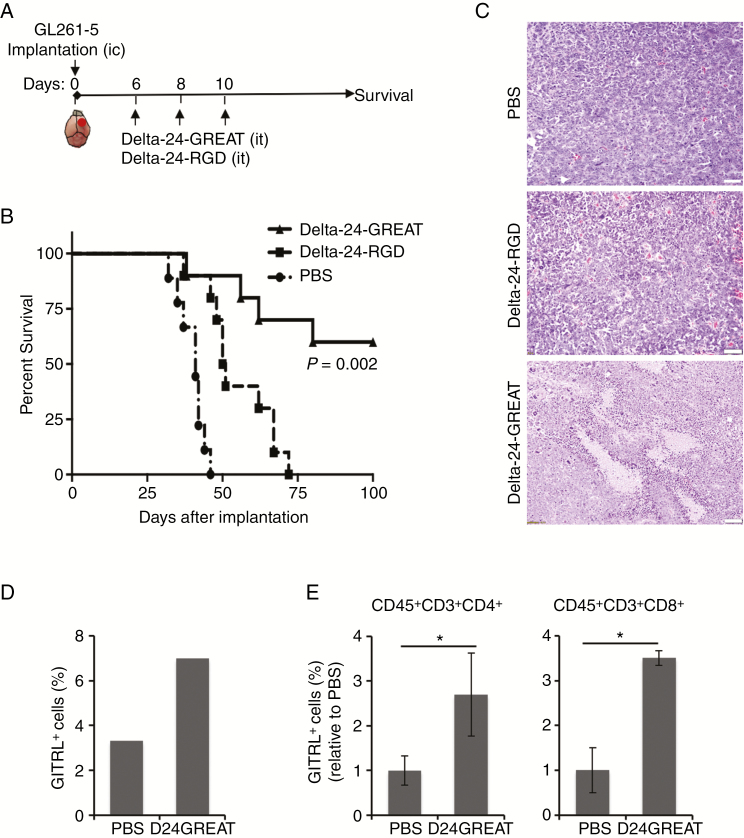
In vivo effect of Delta-24-GREAT. (**A**) Schema of the preclinical study. GL261-5 cells (5 × 10^4^ cells/5 µL) were implanted intracranially (ic) in the right caudate nucleus of C57BL/6 mice using a guide-screw system. Delta-24-GREAT or Delta-24-RGD (5 × 10^7^ pfu; 5 µL) was intratumorally (it) injected on day 6, 8, and 10 after tumor cell implantation. PBS was used as control. (**B**) Survival plots of experiment described in (A) showed prolonged survival with long-term survivors (>100 days) of Delta-24-GREAT-treated mice. (*n* = 10, Delta-24-RGD and Delta-24-GREAT; *n* = 9, PBS). *P* = .002 (log-rank test; Delta-24-GREAT vs Delta-24-RGD). (**C**) Histopathological examination of hematoxylin and eosin stained sections of tumors of mice that showed signs of disease, and displayed high cellularity. Delta-24-GREAT-treated mice showed increased areas of necrosis. Scale bar = 100 μm. (**D**) Expression of mGITRL was analyzed by FACS 15 days after tumor implantation, and 5 days after the last viral dose. Data are represented as percentage of positive cells (3 brains were pooled for analysis). (**E**) Expression of mGITR in CD4+ and CD8+ T cells was analyzed by FACS 15 days after tumor implantation. Data are represented as mean ± SD, relative to PBS values (equal to 1) (*n* = 3). *P < .05, Student’s *t*-test, double sided.

We tested the ability of the armed-Delta-24-RGD in inducing anti-glioma effect in vivo by injecting Delta-24-GREAT into GL261-5-derived intracranial tumors in C57BL/6 mice (Figure 2A). Delta-24-GREAT treatment resulted in prolonged survival of the immunocompetent mice (*P* = .002, vs Delta-24-RGD; log-rank test) with a remarkable difference in the median survival between Delta-24-RGD- and Delta-24-GREAT-treated mice (median survival: 50.5 days vs undefined, respectively). Interestingly, while Delta-24-RGD-treated mice did showed signs of disease by day 37 after cell implantation, and the treatment did not result in any long-term survivor mice, 60% of mice treated with Delta-24-GREAT survived more than 100 days (Figure 2B). Histopathological examination of the tumors collected during the 37–60 days of the experiment from the no long-term survivor mice displayed the presence of extensive necrotic areas in Delta-24-GREAT-treated tumors when compared to Delta-24-RGD- or PBS-treated tumors (Figure 2C; Supplementary Figure 2A). These results illustrate the enhanced anticancer effect of the armed-oncolytic adenovirus Delta-24-GREAT compared to the parental Delta-24-RGD, resulting in a significant number of long-term survivors.

Flow cytometry studies used to detect the expression of mGITRL showed an increase in the number of cells expressing GITRL 5 days after the last intratumoral dose of Delta-24-GREAT (Figure 2D). Of further interest, we also detected an increase in the expression of GITR in cytotoxic T lymphocytes (CTLs; CD45^+^CD3^+^CD8^+^) and helper T cells (Th cells; CD45^+^CD3^+^CD4^+^) (Figure 2E), suggesting the possibility of a costimulatory GITR/GITRL signal in both Th cells and CTLs which, in turn, might facilitate the development of an adaptive immune response.

### Delta-24-GREAT Treatment Results in Enhanced Recruitment and Activation of T cells

**Figure 3.**
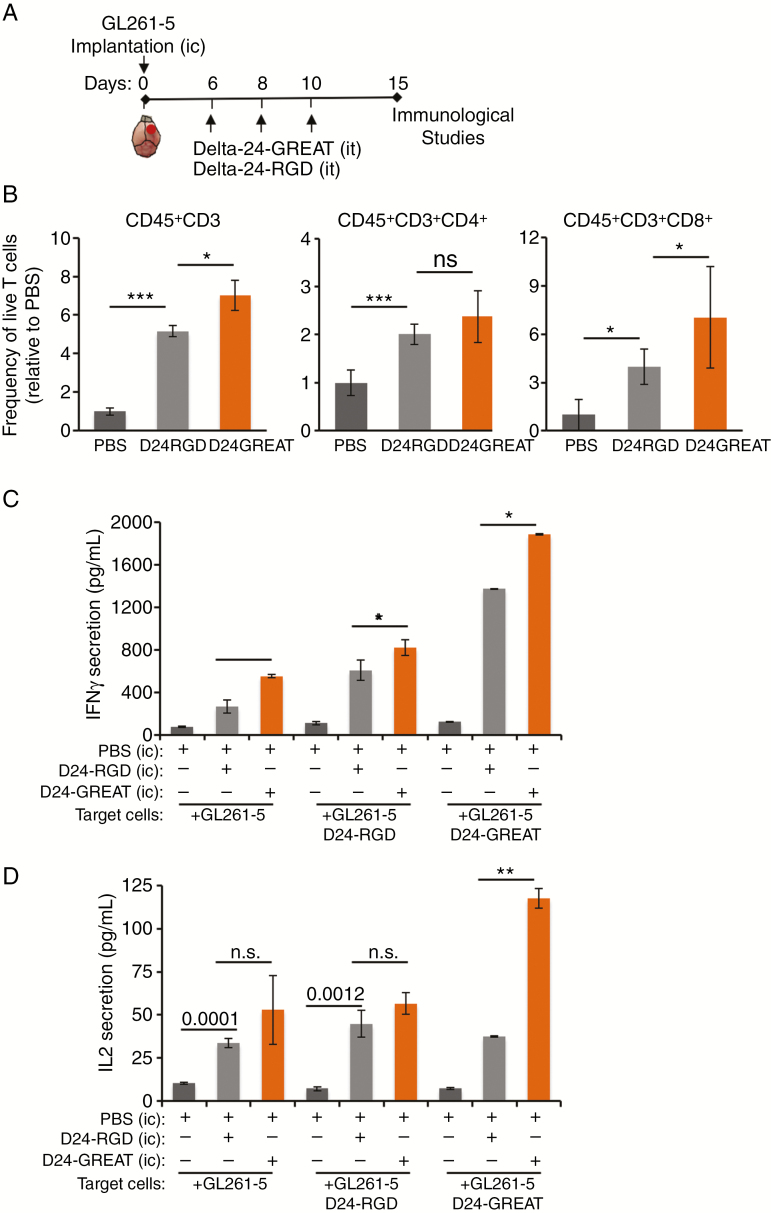
Immune activation upon Delta-24-GREAT treatment. (**A**) Schematic representation of the time point analysis to assess immunological studies. (**B**) Frequency of infiltrating lymphocytes CD45^+^CD3^+^, CD45^+^CD3^+^CD4^+^ (helper T cells), and CD45^+^CD3^+^CD8^+^ (cytotoxic T cells) was analyzed by FACS on day 15 after cell implantation. Data are represented as mean ± SE, relative to PBS (equal to 1). *n* = 3 for CD45^+^CD3^+^; *n* = 6 for CD45^+^CD3^+^CD4^+^ and CD45^+^CD3^+^CD8^+^. *P < .05; ***P < .001, *ns* = not significant (Student’s *t*-test, double sided). (**C** and **D**) Expression of Th1-associated cytokines produced by activated splenocytes from Delta-24-GREAT-treated mice. IFNγ (**C**) and IL-2 (**D**) levels were quantified by ELISA in supernatant of splenocytes obtained from each treated group of GL261-intracranial bearing mice, cocultured for 48 hours with the indicated target cells. Data are represented as mean ± SD. *ns* = not significant; *P < .05; **P < .01; ***P < .001 (Student’s *t*-test, double sided).

To further understand the mechanism related to the anticancer effect of Delta-24-GREAT, we examined phenotypic changes of lymphocyte populations within the tumor after three intratumoral doses of the virus. As expected, Delta-24-RGD treatment resulted in an increase in the frequency of tumor-infiltrating lymphocytes: T lymphocytes (CD45^+^/CD3^+^), Th cells, and CTLs. Of note, Delta-24-GREAT treatment led to a higher percentage of T lymphocytes and CD8^+^ killer T cells than Delta-24-RGD treatment (*P* < .05, Student’s *t*-test) (Figure 3B).

We were also interested in studying functional modifications of T cells after the treatment. To this end, the activity of lymphocyte population was assessed by detecting the secretion of the Th1 cytokines IFN-γ and IL-2 by splenocytes from GL261-5-bearing mice treated with PBS, Delta-24-RGD, or Delta-24-GREAT. These splenocytes were cultured in the presence of uninfected or infected GL261-5 cells. Splenocytes derived from Delta-24-GREAT-treated mice secreted higher levels of IFN-γ than those from both Delta-24-RGD- and PBS-treated mice. Noteworthy, activation of splenocytes in the context of non-infected GL261 cells was enhanced when they were derived from mice previously infected with Delta-24-RGD and this activation was even significantly higher when the mice had been treated with Delta-24-GREAT (*P* < .001, Student’s *t*-test) (Figure 3C). These results suggest that splenocytes are not only activated against viral antigens, but they also recognize tumoral antigens. Expression of IL-2, assessed in parallel experiments, displayed a similar trend, albeit to a lesser degree than IFN-γ expression studies (Figure 3D). Collectively, our data show that glioma treatment with Delta-24-GREAT leads to a higher recruitment of T-cell lymphocytes, especially CTLs, within the tumors than the treatment with the parental Delta-24-RGD. It is noteworthy that functional studies showed that lymphocytes recognized not only viral antigens but also tumoral antigens, suggesting the triggering of antitumoral immunity.

### Delta-24-GREAT Treatment Results in an Antigen-Restricted Antitumor Memory Effect and in the Generation of Central Immune Memory

**Figure 4.**
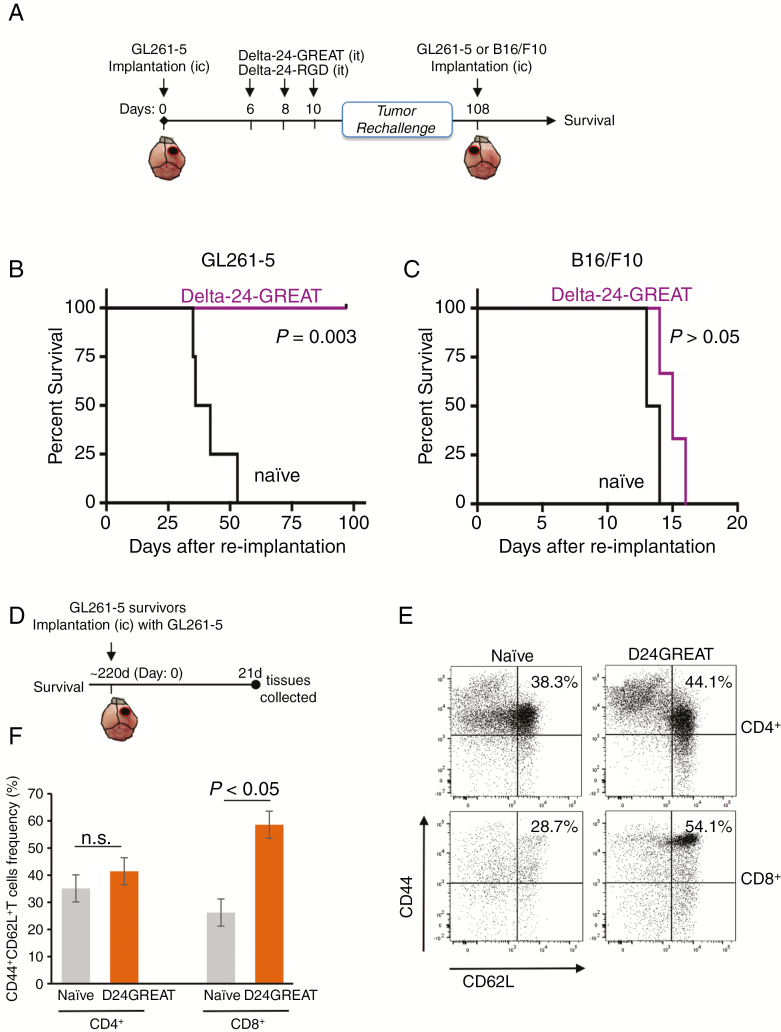
Delta-24-GREAT induced antitumor memory effect and generation of central immune memory. (**A**) Schema of the preclinical rechallenge study. (**B** and **C**) C57BL/6 mice treated with Delta-24-GREAT that survived after GL261-5 intracranial implantation (*n* = 6) were re-challenged with intracranial injection of GL261-5 (**B**) (5 × 10^4^ cells; *n* = 3) or B16/F10 melanoma cells (**C**) (1 × 10^3^ cells; *n* = 3) in their contralateral, left hemisphere. Naïve mice with similar age were used as control (*n* = 3). Data are represented as Kaplan–Meier curves. *P* values are shown (log-rank test). (**D**) Surviving mice from experiment depicted in (B) were intracranially implanted again with GL261-5, and 21 days later cervical and axillary lymph nodes were collected and analyzed by flow cytometry (*n* = 3). (**F** and **E**) Central memory T cells (CD44^+^CD62L^+^) in CD45+CD3+CD4+ and CD45+CD3+CD8+ cells were quantified in lymph nodes from mice treated with Delta-24-GREAT. Naïve mice with similar age (bearing tumor, no treatment) were used (*n* = 3). Data are represented as mean ± SD. ns = not significant; *P < .05, Student’s *t*-test, double sided. (**E**) Representative flow cytometry dot plot of CD44^+^CD62L^+^ central T memory analysis.

To investigate whether the antitumor effect exerted by Delta-24-GREAT resulted in a durable protection, we performed tumor rechallenging experiments. We implanted cancer cells in the brains of naïve and long-term surviving mice (>100 days after glioma implantation) that had been treated previously with Delta-24-GREAT (Figure 4A). These mice were randomly divided into two groups: in the first group, we injected GL261-5 glioma cells, and in the second group, mice were implanted with a heterologous tumor type, B16-F10 melanoma cells (Figure A–C). Although implantation of GL261-5 cells resulted in tumor development in naïve mice (median survival of 39 days), mice treated previously with Delta-24-GREAT did not show any manifestations of disease, and all of them survived more than 100 days (*P* = .003, log rank test) (Figure 4B). On the other hand, implantation of B16-F10 into the brains of naïve mice and previously Delta-24-GREAT-treated mice resulted in tumor development with a similar survival pattern (median survival of 13.5 and 15 days, respectively) (*P* < .05, log-rank test) (Figure 4C).

To further assess the role of an immune protection after Delta-24-GREAT, long-term survivor mice from the aforementioned experiment were rechallenged again with a second injection of GL261-5, and analyzed for central T memory (Figure 4D). Lymph nodes were collected after 21 days of the tumor rechallenge. Naïve mice of similar age were used as controls. Our results showed an increase in central CD8^+^ T memory (CD44^Hi^CD62L^+^) in mice previously treated with Delta-24-GREAT (*P* < .05, Student’s *t*-test), but there were not significant changes in CD4^+^ T central memory when compared to data obtained from naïve mice (Figure 4E and F; Supplementary Figure 2B). These results indicate that the effect of Delta-24-GREAT in the immune response against the tumor is antigen-restricted to the treated tumor. In addition, the development of a CD8^+^ T-cell memory strongly suggests a long-term protection for cancer recurrence.

## Discussion

The translation of the virotherapy to the clinical settings has resulted in encouraging results for the treatment of patients with cancer. Results from three clinical trials using oncolytic viruses to treat patients with recurrent glioblastoma have been recently reported.^4,6,7^ Of interest, these clinical trials resulted in approximately 20% of long-term survivors (>3 y) after a single intratumoral viral injection, following a pseudoprogression process associated with an inflammatory response. This outcome is encouraging and suggests a common and fundamental mechanism underlying these clinical responses. In addition, they also indicated that a significant number of patients did not respond to the oncolytic virotherapy. To increase the percentage of responders to viroimmunotherapy, we decided to arm Delta-24-RGD with a GITRL expression system with the goal of activating the T-cell population recruited to the tumors after viral infection.

Activation of naïve T cells requires two signals: the recognition of the antigen-MHC complexes by the TCR and a costimulatory signal. The latter is provided by the engagement of B7 molecules on the APC with CD28 on the T-cell surface.^17^ However, several challenges are present in cancer, and specifically in gliomas, that prevent this process from happening. First, gliomas are considered “cold tumors” meaning they contain a nonimmunogenic tumor microenvironment^18,19^; and second, the tumor microenvironment is often characterized by the presence of anergic T cells, due to the lack of positive costimulatory molecules on the surface of cancer cells.^17^ Using oncolytic adenoviruses in preclinical and clinical studies, we have observed the presence of an inflammatory response associated with tumor cell death and the recruitment of T cells into the tumor microenvironment.^4,13^ This inflammatory setting should favor the antigen presentation process by APCs. In the work presented here, we showed that treatment with Delta-24-GREAT resulted in a further enhanced presence of CD3^+^ T lymphocytes and CTLs (CD8^+^) when compared to the effect of Delta-24-RGD, suggesting the possibility of a stronger immune response.

A second barrier to an effective antitumor immunity relates to the lack of expression of costimulatory molecules in cancer cells.^17^ To overcome this intrinsic tumor challenge, we armed Delta-24-RGD to express GITRL on the surface of infected cancer cells, where it will be able to engage with GITR molecules present on the immune cells. In our study, infection of a panel of murine and human glioma cells, including glioma stem cells, with Delta-24-GREAT resulted in expression of GITRL in more than 70% of the cells in vitro, and exogenous GITRL was detected in vivo after intratumoral injection. Of interest, we observed an increase in the expression levels of GITR in the tumor-infiltrating lymphocytes, CD4^+^ and CD8^+^ T cells, two weeks after Delta-24-GREAT treatment. The availability of increased levels of GITR and GITRL within the tumor microenvironment together with an increase of CTLs should favor the development of an adaptive immune response.

It has been reported that GITR ligation protects T cells from activation-induced cell death leading to an increase in memory cells.^11,20,21^ In agreement with this theory, we observed that Delta-24-GREAT treatment resulted in the development of a systemic immune response as demonstrated by resistance of long-term survivor mice to tumor rechallenge experiments accompanied by an increased CD8^+^ T central memory. These results suggest that Delta-24-GREAT treatment is generating an antitumor immune memory, with the potential of challenging tumor recurrence and with curative potential. Although out of the scope of this study, the mechanisms by which GITR/GITRL induces anticancer effect might include a role in reversing both T-cell anergy^22^ and CD8+ cells exhaustion.^23^ Currently, the GITR/GITRL pathway is being targeted in the clinic to treat adult patients with advanced solid tumors by using traditional bivalent antibodies and multivalent GITR fusion proteins.^12^ Brain tumors present several challenges for this mode of treatment, from being located behind the blood-brain barrier with difficult access to antibodies to having a low presence of immune cells to be targeted by these agents.^24^ Direct intratumoral injection of a GITRL-armed oncolytic adenovirus overcomes some of these challenges, by increasing the frequency of T-cell population within the tumor, and by inducing T-cell activation due to expression of GITRL. Therefore, Delta-24-GREAT might be a feasible strategy for the treatment of gliomas.

The members of the TNFR family of proteins sustain T-cell responses after initial T-cell activation. The effects of these costimulatory molecules such as GITR, 4-1BB, and OX40 seem to be non-redundant and may have independent functions with respect to CD4 and CD8 T-cell activation, as suggested by analysis of mice lacking both OX40L and 4-1BBL.^25,26^ Therefore, it might be possible to design combinations of these T-cell activators in order to gain a synergistic anticancer effect. To this end, we have recently reported the anti-glioma effect of an oncolytic adenovirus expressing OX40L^27^ and we plan to test the best combinations of TNFR molecules in future studies.

This study encourages the preclinical testing of the next generation of oncolytic viruses armed with regulators of the immune checkpoints. This vertical improvement in the virotherapy design seems to be associated with an enhanced immune response against the tumor and the establishment of central immune memory, suggesting the possibility of improving the response rate to virotherapy in future clinical trials.

## Supplementary Material

vdz009_suppl_Supplementary_Figure_1

vdz009_suppl_Supplementary_Figure_2

vdz009_suppl_Supplementary_Figure_Legend
