# Supplementary figures and images for: GITRL-armed Delta-24-RGD oncolytic adenovirus prolongs survival and induces anti-glioma immune memory
Source: Neurooncol Adv. 2019 Jun 5;1(1):vdz009. doi: 10.1093/noajnl/vdz009 (PMC6777503; doi:10.1093/noajnl/vdz009)

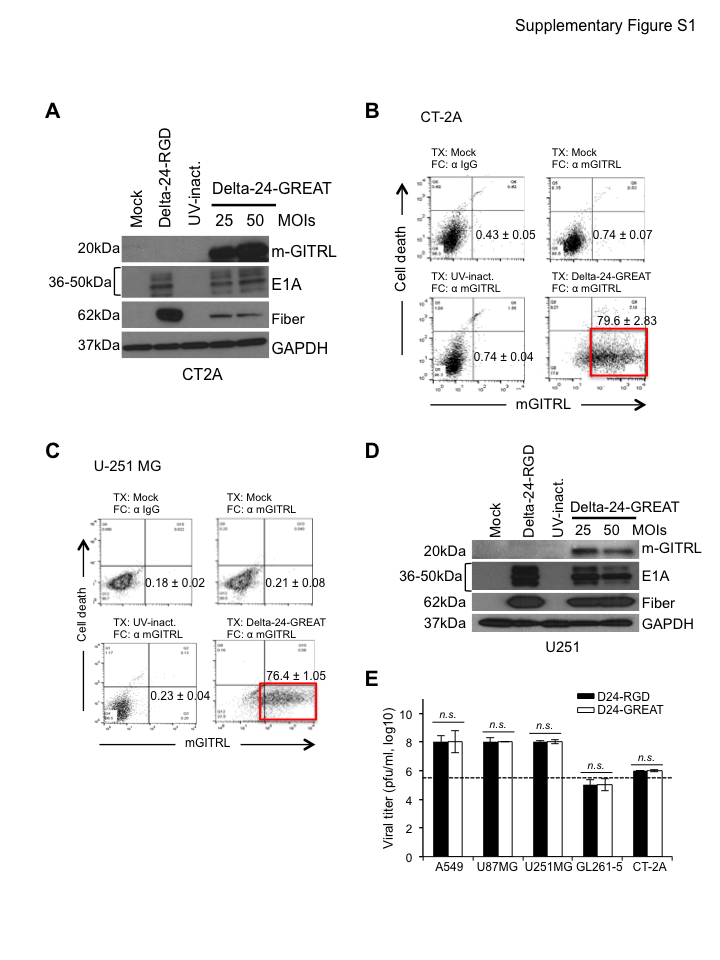

Supplement: vdz009_suppl_Supplementary_Figure_1 [file vdz009_suppl_supplementary_figure_1.png]

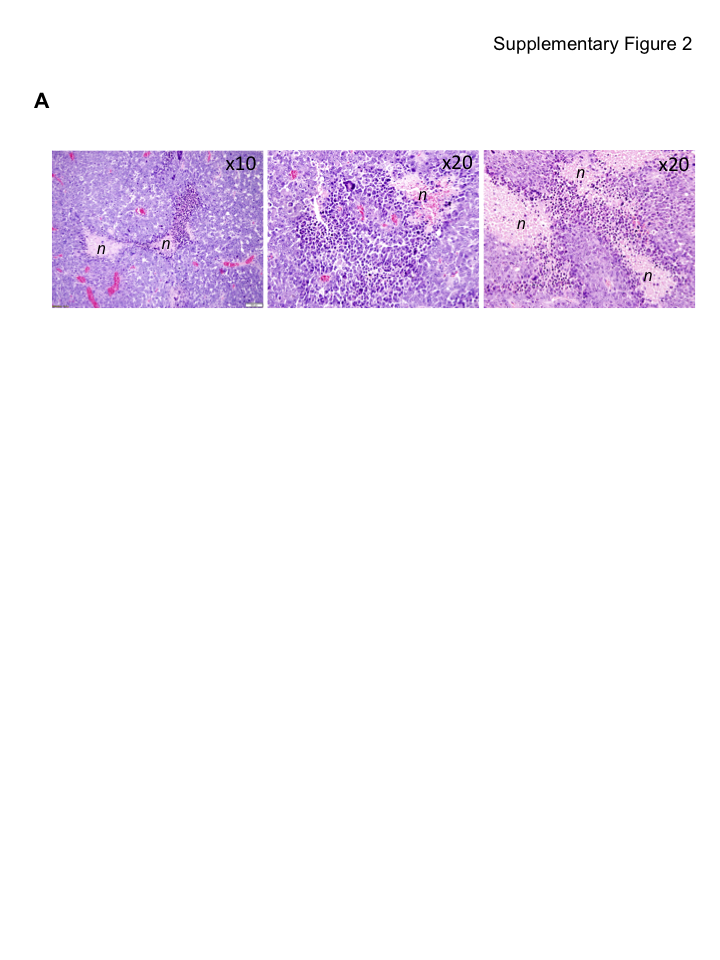

Supplement: vdz009_suppl_Supplementary_Figure_2 [file vdz009_suppl_supplementary_figure_2.png]
