# Supplementary material for: GITRL-armed Delta-24-RGD oncolytic adenovirus prolongs survival and induces anti-glioma immune memory
Source: Neurooncol Adv. 2019 Jun 5;1(1):vdz009. doi: 10.1093/noajnl/vdz009 (PMC6777503; doi:10.1093/noajnl/vdz009)
Supplement: vdz009_suppl_Supplementary_Figure_Legend [file vdz009_suppl_supplementary_figure_legend.docx]

**SUPPLEMENTARY FIGURE LEGENDS**

**Supplementary Figure 1. Additional information on infectivity and replication of Delta-24-GREAT in murine and human glioma cells. A** and **D)** Expression of viral proteins, E1A and fiber, and mGITRL in the murine glioma CT-2A cells (**A**) and human glioma U251 MG cells (**D**), 48 hours after infection with Delta-24-GREAT assessed by Western blot. UV-inactivated Delta-24-GREAT and Delta-24-RGD were used as controls (50 MOIs). GAPDH was used as loading control. **B** and **C)** Expression *in vitro* of the murine GITR ligand on the surface of Delta-24-GREAT-infected murine glioma cells CT-2A (100 MOIs) (**B**) and human glioma cells U-251MG (50 MOIs) (**C**). Mock-infected cells stained with IgG isotype as primary antibody and UV-inactivated Delta-24-GREAT-infected cells were used as control. Non-viable cells were excluded from the analysis using ethidium homodimer. Data are showed as mean ± SD of three independent experiments. α, antibody. **E)** Replication ability of Delta-24-GREAT in a panel of human and murine cell lines 48 hours after infection (10 MOIs). Data are shown as mean ± SD of three experiments; *ns* = not significant, Student’s test. Dashed line, initial viral dose.

**Supplementary Figure 2. Additional information on anti-cancer effect of Delta-24-GREAT. A)** Hematoxylin and eosin-stained sections of tumors collected from mice showing signs of disease from experiment depicted in Figure 2A. Tumor infiltrating lymphocytes are observed surrounding necrotic areas (*n*) upon Delta-24-GREAT intracranial treatment.
